# A Novel FLI1 Monoclonal Antibody Which Recognizes EWS::FLI1 with High Affinity Is Useful for Detecting Ewing Sarcoma

**DOI:** 10.3390/antib14040097

**Published:** 2025-11-10

**Authors:** Saravana P. Selvanathan, Olivia O. Lansinger, David V. Allegakoen, Emma J. W. McGuire, Ashley R. Gaffey, Jeff R. Petro, Purushottam B. Tiwari, Quinn Tufiño, Aykut Üren, Jeffrey A. Toretsky

**Affiliations:** 1 Departments of Oncology and Pediatrics, Georgetown University, Washington, DC 20057, USA; 2 Departments of Oncology, Georgetown University, Washington, DC 20057, USA

**Keywords:** monoclonal antibody, FLI1, EWS::FLI1, ewing sarcoma, surface plasmon resonance (SPR)

## Abstract

Background: Ewing sarcoma (ES) is a rare tumor that affects children, adolescents, and young adults. ES is associated with high morbidity in all patients and high mortality for those who present with metastatic disease. A chromosomal translocation, either t(11;22)(q24;p12) or t(21;22)(q22;q12) leads to the fusion oncoproteins EWS::FLI1 or EWS::ERG in 95% of ES patients. We recognized a critical need for a stably sourced high-affinity antibody that recognizes EWS::FLI1 with maximal specificity. Understanding EWS::FLI1 protein complexes is a pivotal gap in ES knowledge that necessitates the development of antibodies capable of identifying native proteins in solution. Further, variable epitope sequencing of a monoclonal antibody enables the construction of degraders and nanobody identifiers. Methods: Monoclonal antibodies were produced following informed peptide synthesis, injection, and hybridoma creation. Hybridoma antibodies were validated for specificity and function. Results: Our results indicate that the FLI1 1.2 monoclonal antibody, which recognizes the EWS::FLI1 fusion oncoprotein, can be reliably applied to multiple molecular biology applications like immunoblot, immunoprecipitation, immunofluorescence, and immunohistochemistry. This FLI1 1.2 monoclonal antibody has a high affinity of 0.3 nM KD to EWS::FLI1. In terms of specificity, this antibody is highly specific to EWS::FLI1 and some cross reactivity with ERG. Conclusions: This reagent will provide the research community with valuable tools for further biochemical and genomic interrogation of the oncogenic activity of EWS::FLI1 in ES.

## 1. Introduction

FLI1 is a member of the ETS (E-twenty-six-specific sequence, or E26 transforming sequence) family of transcription factors characterized by a highly conserved DNA-binding domain [1]. The ETS family of transcription factors contains 28 genes in humans, which are a functionally heterogeneous group of gene regulators that share a structurally conserved, winged helix-turn-helix DNA-binding domain (DBD), also known as the ETS domain [2]. FLI1’s biologic role is altered in Ewing sarcoma (ES), a rare tumor that affects children, adolescents, and young adults. ES has a high morbidity in all patients, and for those who present with metastatic disease, a high mortality [3,4]. A chromosomal translocation, either t(11;22)(q24;p12) or t(21;22)(q22;q12), leads to the fusion oncoproteins EWS::FLI1 or EWS::ERG, respectively, in 95% of ES patients [5]. These fusion proteins are critical initiators and maintenance proteins in ES tumors. EWS::FLI1 is known to alter transcription and splicing, which are critical for cellular transformation [6,7,8,9]. Furthermore, these fusion proteins are uniquely present in tumors and absent in non-cancerous tissue, making them ideal therapeutic targets due to their specific presence and vital nature in ES.

Investigating EWS::FLI1’s role in biological processes as well as understanding the protein–protein interactions required for its function is necessary for the development of new therapeutic interventions targeting the protein. Many biological experiments that investigate EWS::FLI1 require a high-affinity antibody, including immunoblotting, immunohistochemistry, immunoprecipitation, and chromatin immunoprecipitation [10]. ES cells do not express wild-type (WT) FLI1, hence the FLI1 antibodies are used in research to study the localization of the EWS::FLI1 protein in cells and tissues, as well as its interactions with other proteins and signaling pathways [11,12]. The EWS antibody recognizes both WT EWSR1 and the EWS::FLI1 fusion protein. Elucidating the biophysical interactions of EWS::FLI1 and its protein partners is key for understanding its functions [13]. Detection of native proteins adds significant validity to conclusions about ES biology.

Aside from its critical role in recognizing and identifying the EWS::FLI1 fusion oncoprotein in ES, FLI1 antibodies have also been used as a diagnostic tool [14,15]. In addition, targeting EWS::FLI1 using a FLI1 antibody may have therapeutic potential in ES. For instance, FLI1 antibody binding enables the targeted protein degradation of EWS::FLI1, a key avenue for developing therapeutic design [16,17].

Polyclonal FLI1 antibodies were often useful but limited by the production in rabbits or other animals. Until now, monoclonal FLI1 antibodies derived from a renewable hybridoma have not been commercially available. We created a human monoclonal antibody synthesized in a renewable hybridoma that is useful in various assays including, but not limited to, immunoblotting, immunoprecipitation, immunofluorescence, and antibody-mediated protein degradation. This report demonstrates a novel FLI1 antibody which recognizes the ES fusion-specific EWS::FLI1 with high affinity and specificity that can be reliably applied to multiple molecular biology applications.

## 2. Materials and Methods

### 2.1. Antibody Development

**Figure 1:**
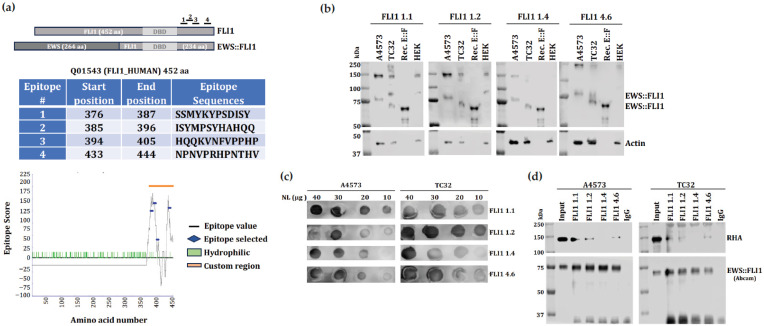
Hybridoma screening leads to novel antibody binding to EWS::FLI1. (**a**) Epitope locations on the FLI1 aligned with EWS::FLI1 (**top**). Using a sequence algorithm for predicting antigenicity we selected four 12-residue fragments to generate monoclonal antibodies. The amino acid numbers correspond to the full length FLI1 (**middle**). Likely antigenicity epitope score for each peptide was shown on the graph (**bottom**). (**b**) Immunoblot analysis showing selected custom FLI1 monoclonal antibody (1:1000 dilution) detection of EWS::FLI1 fusion protein (15 μg of total protein) in the ES cell line TC32 (type 1) and A4573 (type 3), purified recombinant EWS::FLI1 fusion protein (300 ng), and HEK293 (lack EWS::FLI1). Actin used as a loading control. (**c**) Dot blot analysis showing titration of selected FLI1 monoclonal antibody (1:1000 dilution) used against different concentration of nuclear lysates (NL) from A4573 and TC32. (**d**) Nuclear co-immunoprecipitation (co-IP) of EWS::FLI1 screening using selected FLI1 antibody clones. Immunoprecipitated RHA is shown. An amount of 10% of total nuclear lysate was used as input. Immunoblot detection of RHA and EWS::FLI1 (Abcam, Waltham, MA, USA, Cat. No. ab15289, 1:1000 dilution).

A mouse monoclonal antibody (clone 3C16–FLI1 1.2) was produced (Abmart, Inc., Shanghai, China) by immunization of 6 to 8-week-old female BALB/c mice with a synthetic antigenic peptide containing amino acid residues corresponding to the C-terminal end after the DNA-binding domain (AA 376 to 387-SSMYKYPSDISY) of FLI1 (Figure 1a). Three additional peptides were also immunized (Figure 1a). The procedures for care and use of animals were approved by the Abmart Institutional Animal Care and Use Committee (IACUC) [18]. Anti-serum screening (ELISA) was taken after the final immunization and 2 out of 3 mice with the best titer were executed and total splenocytes were isolated for cell fusion with SP2/0 cells. Each fusion was diluted into several 384 plates and 2 rounds of sub-cloning would be performed; meanwhile, single peptide ELISA binding test would be performed to validate the binding property of each clone. Selected hybridoma cells were transferred to lager dishes for both storage and antibody production. Hybridoma cells were injected into mice (i.p.) for ascites generation.

### 2.2. Hybridoma Cell Culture

Hybridoma cells were grown in DMEM with 15% FBS at 37 °C in 5% CO_2_. Healthy expansion cells were split at 60% confluence. Hybridoma cells were removed from the plate by gentle pipetting and did not require trypsin digestion. The media was changed 2–3 times per week. Supernatants were collected 12 h after the cell density reached 1 × 10^6^/mL [19].

### 2.3. Cell Lines and Reagents

ES cell lines TC32 and A4573 were originally obtained from the NIH repository in 1982, and they were grown in RPMI with 10% FBS and 1% HEPES. All cell lines were grown at 37 °C in 5% CO_2_ and passaged every 2–4 days. Cell line integrity was confirmed by fingerprinting. The genetic information of the cell lines was available in Cellosaurus. Cell lines were tested for mycoplasma in domo at regular intervals.

### 2.4. Nuclear Co-Immunoprecipitation

Co-immunoprecipitation (Co-IP) was performed to validate EWS::FLI1 complex protein–protein interactions. The Nuclear Complex Co-IP Kit (Active Motif, Carlsbad, CA, USA; Cat. No. 54001) was used to prepare nuclear extract from TC32 and A4573 cells according to the manufacturer’s instructions. The protein G magnetic beads were used for Co-IP, and the IP was performed on 600 μg samples using 2 μg of custom FLI1 monoclonal antibody and mouse monoclonal IgG (as a negative control). Immunoblots were performed individually using the following antibodies: RHA (Everest Biotech, Newark, CA, USA; Cat. No. EB09297), PRPF6 (Abcam, Waltham, MA, USA; Cat. No. ab99292), SFPQ (Abcam, Waltham, MA, USA; Cat. No. ab99357), DDX5 (Abcam, Waltham, MA, USA; Cat. No. ab21696), hnRNPK (Abcam, Waltham, MA, USA; Cat. No. ab18195), SRSF3 (Abcam, Waltham, MA, USA; Cat. No. ab73891), and SRSF9 (Abcam, Waltham, MA, USA; Cat. No. ab236414). Detection was carried out using Millipore Immobilon Western Chemiluminescent HRP Substrate per the manufacturer’s instructions (Millipore Sigma, Rockville, MD, USA) using a Li-COR Odyssey FC Imaging System.

### 2.5. Immunoblot

Samples were subjected to SDS-PAGE and then transferred to an Immobilon-P membrane (Millipore, Billerica, MA, USA). Non-specific binding sites were blocked upon incubation in 5% nonfat dry milk diluted in 1X TTBS (20 mM Tris-HCl, pH 7.5, 150 mM NaCl, 0.05% Tween 20) for 1 h at room temperature (RT). The membranes were then incubated with primary antibodies for 2 h at RT or overnight at 4 °C, according to the manufacturer’s recommendations. For FLI1 1.2 monoclonal antibody, the membranes were incubated for 2 h at RT. Dilution for primary antibodies corresponding to FLI1, PRPF6, RHA, SFPQ, hnRNPK, and SRSF3 occurred at 1:1000 in 5% BSA diluted in 1X TTBS. Following primary antibody incubation, the membranes were rinsed four times with 1X TTBS in 15 min intervals. Secondary antibody incubation then took place at RT for 1 h. HRP-conjugated anti-rabbit (GE Healthcare Bio Sciences, Pittsburgh, PA, USA; Cat. No. NA934V) and anti-mouse (GE Healthcare Bio Sciences, Pittsburgh, PA, USA; Cat. No. NA931V) secondary antibodies were diluted at 1:5000 in 1X TTBS. HRP-conjugated anti-goat secondary antibody (Santa Cruz Biotechnology, Dallas, TX, USA; Cat. No. sc-2354) was diluted at 1:3000 in 5% nonfat dry milk in 1X TTBS. Anti-actin-HRP (Abcam, Waltham, MA, USA, Cat. No. ab49900) was diluted at 1:15,000 in 5% nonfat dry milk in 1X TTBS and incubated with membranes for 30 min at RT. Following either anti-actin or secondary antibody incubation, blots were washed four times in 1X TTBS in 15 min intervals, and then developed using Millipore Immobilon Western chemiluminescent HRP substrate according to the manufacturer’s instructions (Millipore Corporation, Billerica, MA, USA) using a Li-COR Odyssey FC Imaging System.

### 2.6. Dot Blot

Nuclear lysates of ES were spotted directly onto a nitrocellulose membrane using circular templates. Using a narrow-mouth pipette tip, 2 μL of the samples were spotted onto the nitrocellulose membrane at the center of the grid. The area that the solution penetrated was minimized (usually 3–4 mm in diameter) by applying it slowly and letting the membrane dry. Non-specific binding sites were blocked upon incubation in 5% nonfat dry milk diluted in 1X TTBS (20 mM Tris-HCl, pH 7.5, 150 mM NaCl, 0.05% Tween 20) for 1 h at RT. The membranes were incubated with primary antibodies diluted at 1:1000 in 5% BSA in 1X TTBS at RT for 2 h. After rinsing three times with 1X TTBS, the membranes were incubated for 1 h at RT in HRP-conjugated anti-mouse (GE Healthcare Bio Sciences, Pittsburgh, PA, USA; Cat. No. NA931V) secondary antibody diluted at 1:5000 in 1X TTBS. The blots were then washed three times in 1X TTBS and then developed using Millipore Immobilon Western chemiluminescent HRP substrate according to the manufacturer’s instructions (Millipore Corporation, Billerica, MA, USA). Chemiluminescence was detected using a Li-COR Odyssey FC imaging system.

### 2.7. Surface Plasmon Resonance (SPR)

SPR experiments were performed using a Biacore T200 SPR instrument (Cytiva, Marlborough, MA, USA) with a CM5 chip at 25 °C and data were evaluated using the Biacore T200 evaluation software version 3.2.1 [20]. Anti-FLI1 1.2 antibody was used as the ligand to immobilize on the CM5 chip using standard amine coupling chemistry to a level of 3000–4400 RU in the presence of 10 mM sodium acetate buffer at pH 5.5. HBS-P (10 mM HEPES pH 7.4, 150 mM NaCl, 0.05% surfactant P20) was used as the immobilization running buffer. EWS::FLI1, peptide-1, and peptide-4 were used as analytes to flow over the ligand-immobilized surface in kinetics experiments. In the blocking experiments, 100 nM EWS::FLI1 was injected as an analyte in the absence and presence of peptide-1 or peptide-4 captured on the antibody surface. An amount of 1000 nM of either peptide was injected for at least 90 s prior to injecting EWS::FLI1. HBS-P supplemented with 1% (*v*/*v*) DMSO, and 0.1% (*v*/*v*) glycerol was used as the running buffer during both kinetics and blocking experiments. The flow rate of all analyte solutions was maintained at 50 μL/min. Two 20 s pulses of 50 mM NaOH were injected for surface regeneration. SPR sensorgrams obtained for analysis were both reference (corresponding to the reference flow cell) and blank (buffer only) subtracted. We performed statistical analysis using GraphPad Prism (Version 10) with Student’s *t*-test; data are expressed as means ± SD from three different experiments.

### 2.8. Immunofluorescence (IF)

Cells were seeded at 100,000 cells per well in 12-well plates on sterilized, collagenized coverslips. After 24 h, the cells were fixed in 10% formalin for 5 min and then washed with Phosphate-Buffered Saline (PBS). Cells were then treated with 0.5% Triton X-100 for 15 min to permeabilize the nuclei and washed 3 times with PBS. An amount of 5% Bovine Serum Albumin (BSA) was added for a 1 h incubation to eliminate non-specific binding, and a signal enhancer was added for 30 min to reduce charge-linked fluorescent background. Primary antibodies were diluted in 0.5% BSA and incubated with coverslips overnight. Cells subsequently underwent 3 PBS rinses followed by a 1 h incubation with fluorophore-conjugated secondary antibodies protected from light. The coverslips were then rinsed with PBS and treated with 300 nM DAPI solution for 5 min, followed by mounting with Diamond Prolong Antifade Mountant onto glass slides. After curing for 48 h, the slides were imaged at 60X magnification with immersion oil on a Nikon SoRa Spinning Disk Microscope under the following conditions: DAPI nuclear counterstain (λex 350, λem 470), mouse monoclonal EWS::FLI1 (Anti-FLI1 1.2)-goat anti-mouse AlexaFluor 488 (λex 490, λem 525), rabbit monoclonal hnRNPK (Abcam, Waltham, MA, USA, Cat. No. 52600)-goat anti-rabbit AlexaFluor 594 (λex 590, λem 617). The images were processed with denoise and deconvolution NIS Elements (Version 5.41.01) software. Colocalization analysis was performed using BIOP JACoP (Bioimaging and Optics Platform/Just Another Colocalization Plugin) through ImageJ2 (Version 2.14.0/1.54f) [21]. Pearson’s Correlation Coefficient (PCC) is used to quantify colocalization by subtracting the mean intensity from each pixel’s intensity value. A PCC of 1 indicates strong colocalization, while a PCC of 0 indicates no colocalization. PCC is independent of signal levels and signal offset (background).

### 2.9. Variable Region Sequencing

RNA was isolated from hybridoma clone FLI1 1.2 using a Qiagen RNeasy mini kit (Qiagen, Germantown, MD, USA; Cat. No. 74106) with on-column DNase digestion (Qiagen, Germantown, MD, USA; Cat. No. 79256). Reverse transcription and PCR of antibody variable regions using a previously published protocol [22]. Briefly, reverse transcription was performed using an SMARTScribe Reverse Transcriptase (Takara, San Jose, CA, USA; Cat. No. 639536) with a universal template-switching oligonucleotide and specific reverse primers corresponding to heavy, kappa, and lambda chains. Further amplification was performed using a universal forward PCR primer and specific reverse primers for each chain type. PCR products were run on a 1% agarose gel. Amplicons were purified using a Thermo Scientific GeneJET Gel Extraction kit (Thermo Fisher, Carlsbad, CA, USA; Cat. No. K0692) and sequenced on an Oxford Nanopore. Variable region sequences were annotated using the find Antibodies Python script (February 2019 final version) provided in the mentioned protocol.

### 2.10. Modeling of Heavy and Light Chains in Complex with FLI1 Epitope

The heavy and light chain sequences were input into the AlphaFold3 server [23] along with the amino acid sequence of the peptide used as the immunogen.

### 2.11. Cloning of Single-Chain Variable Fragment

Heavy and light chain sequences were codon optimized for expression in human cell lines and synthesized as fragments by Twist Bioscience. Fragments were cloned into a pcDNA3.1 expression vector. Heavy and light chains were linked together with a flexible (GGGGS)_3_ sequence to create a single-chain variable fragment (scFv) construct. This scFv was then fused to the CHIP∆TPR E3 ligase fragment with a GSGSG linker and C-terminal FLAG and 6xHis tags to generate a degrader [24]. An N-terminal 3xFLAG tag was added to increase activity in cells [25].

### 2.12. Immunohistochemistry (IHC)

Immunohistochemical staining of mouse tissues was performed for FLI1 1.2 monoclonal antibody. Five-micron sections from formalin-fixed paraffin-embedded tissues were deparaffinized with xylenes and rehydrated through a graded alcohol series. Heat-induced epitope retrieval (HIER) was performed by immersing the tissue sections at 98 °C for 20 min in Dako LowPH solution (Agilent, Santa Clara, CA, USA; Cat. No. K8005). Immunohistochemical staining was performed using an HRP-labeled polymer from Dako (Agilent, Santa Clara, CA, USA; Cat. No. K4003) according to the manufacturer’s instructions. Briefly, slides were treated with 3% hydrogen peroxide and 10% normal goat serum for 10 min each, and then exposed to primary antibodies for FLI1 commercial (1/100, Abcam, Waltham, MA, USA, Cat. No. ab15289) and the custom FLI1 1.2 monoclonal antibody for 1 h at room temperature. Antibody slides were exposed to the appropriate HRP-labeled polymer for 30 min and DAB chromagen (Dako, Agilent, Santa Clara, CA, USA) for 5 min. Slides were counterstained with Hematoxylin (Fisher, Harris Modified Hematoxylin; Fisher Scientific, Frederick, MD, USA; Cat. No. SH30-4D), blued in 1% ammonium hydroxide, dehydrated, and mounted with Acrymount. Consecutive sections with the primary antibody omitted were used as negative controls. The wash buffer used was 1XTBS with 0.05% Tween 20 (Fisher Scientific, Frederick, MD, USA; Cat. No. AAJ62554AK).

## 3. Results

### 3.1. Selected Hybridoma Titration and Antibody Binding to EWS::FLI1

We chose to create an antibody targeting the FLI1 portion of EWS::FLI1 because EWSR1 is ubiquitously expressed, while FLI1 has much more restricted expression in ES [26,27,28,29,30]. Thus, we used an antigenicity algorithm focused on the amino acid sequence of the FLI1 portion of EWS::FLI1 (Figure 1a). An epitope score was assigned to each residue based on structural features, sequence conservation, hydrophobicity, and solvent exposure. The 12-residue fragments with the highest overall scores were selected as epitopes to generate monoclonal antibodies and are listed along with their start and end positions and corresponding epitope scores in Figure 1a. Twenty-four hybridoma clones were generated based on four independent peptide sequences (Figure S1a). Preliminary hybridoma screening was conducted by ELISA, and four hybridomas were selected with either high titers or sequence characteristics. Ascites from four mice corresponding to FLI1 clones 1.1, 1.2, 1.4, and 4.6 were further screened by immunoblotting for their ability to detect the 75 kDa EWS::FLI1 type 3 protein in A4573 or the 68 kDa EWS::FLI1 type 1 protein in TC32. Recombinant full-length EWS::FLI1 was used as a positive control, and HEK293 cells were used as a negative control (Figure 1b). Dot blots using ES nuclear lysates from A4573 and TC32 showed FLI1 1.1 and 1.2 with the highest titers (Figure 1c). Since a key goal of creating a new antibody was the property of co-immunoprecipitation (Co-IP), a Co-IP of EWS::FLI1 was performed, followed by immunoblotting for its direct binding partner RNA Helicase A (RHA) [10,31]. FLI1 1.1, 1.2, and 4.6 demonstrated Co-IP activity (Figure 1d). We chose FLI1 1.2 antibody since FLI1 1.1 shows additional non-specific bands at 80kDa and 200kDa in TC32 ES cells (Figure 1b). In addition, the FLI1 1.1 hybridoma arrived contaminated during the initial shipment and was then delayed due to COVID-19. Hence, we chose to proceed with FLI1 1.2 for large-scale preparation and immunopurification.

### 3.2. Surface Plasmon Resonance (SPR) Confirms Antibody Specificity to Immunizing Peptide

**Figure 2:**
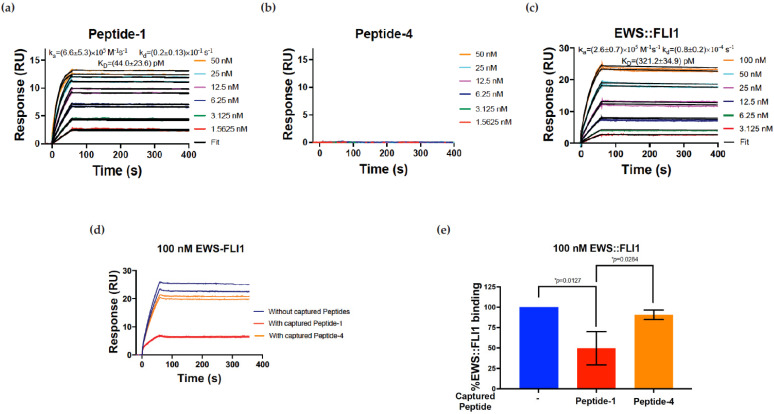
Surface plasmon resonance (SPR) supports antibody specificity using peptide competition. For (**a**–**c**), antibody FLI1 1.2 was immobilized on the surface of an SPR chip. (**a**) Peptide-1, used to generate clones 1.1–1.6 binding to FLI1 1.2. (**b**) Peptide-4, used to generate clones 4.1–4.7 binding to FLI1 1.2. (**c**) Recombinant EWS::FLI1 binding to antibody FLI1 1.2. For (**a**–**c**), colored lines are incremental titration data and black lines are fit to the 1:1 kinetics binding model. The values are mean ± s.d. from three independent experiments. (**d**) Competition assay showing the SPR sensorgram for 100 nM EWS::FLI1 binding to immobilized FLI1 1.2 monoclonal antibody in the absence (blue) and presence of either peptide-1 (red) or -4 (yellow). When EWS::FLI1 binding was tested in the presence of peptides, the peptides were injected into the antibody immobilized surface prior to injecting EWS::FLI1. Each concentration of all samples was injected in duplicates for technical reproducibility. (**e**) Quantified EWS::FLI1 binding competition from (**d**) based on 100% binding in the absence of any peptides calculated using response values at the end of analyte injections (60 s). The values are mean ± s.d. from three independent experiments. * *p* < 0.05.

Immunopurified FLI1 1.2 monoclonal antibody was immobilized on the SPR chip using standard amine coupling chemistry. Peptide-1 or peptide-4 were injected as analytes to measure binding affinity. Peptide-1 bound to FLI1 1.2 with a K_D_ of 44.0 ± 23.6 pM (Figure 2a) and the association (k_on_ or k_a_) and dissociation (k_off_ or k_d_) rate constants of (6.6 ± 5.3) × 10^5^ M^−1^s^−1^ and (0.2 ± 0.1) × 10^−1^ s^−1^, respectively, while peptide-4 (Figure 2b) did not bind to the immobilized antibody. Full-length recombinant EWS::FLI1 bound to immobilized antibody with a K_D_ of 321.2 ± 34.9 pM (Figure 2c) and k_a_ and k_d_ values of (2.6 ± 0.7) × 10^5^ M^−1^s^−1^ and (0.8 ± 0.2) × 10^−1^ s^−1^, respectively. Peptide competition was demonstrated with peptide-1; however, peptide-4 did not show any meaningful competition (Figure 2d). The response values from (2d) were quantified as percentage EWS::FLI1 binding competition based on 100% binding in the absence of any peptides, as shown in Figure 2e. The percentage binding was calculated using response values at the end of analyte injections (60 s). The values are mean ± s.d. from three independent experiments. The results indicated a significant competition to binding using peptide-1 with loss of signal, whereas peptide-4, the control sequence not used in creating FLI1 1.2, did not show loss of binding (Figure 2e).

### 3.3. AlphaFold3 Model to Predicts Structure of Heavy and Light Chains in Complex with FLI1 Epitope

Hybridoma DNA was extracted and sequenced to create a model of the variable epitope region of FLI1 1.2, a finding that indicated it was an IgG kappa light chain clone (Figure S2a). An AlphaFold3-generated model shows a reasonable prediction for the coordinated binding of the peptide (Figure S2b).

### 3.4. FLI1 1.2 Demonstrates Binding to EWS::FLI1 with Relative Specificity

**Figure 3:**
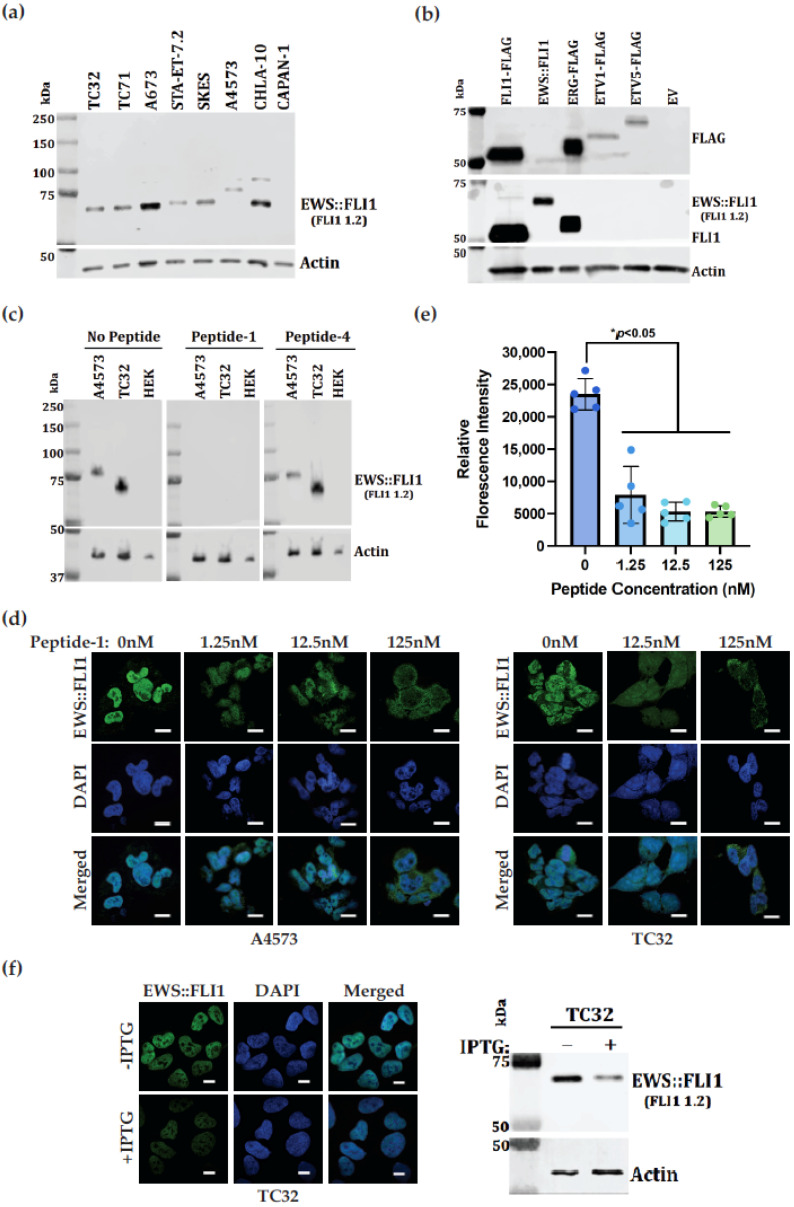
Novel FLI1 monoclonal antibody recognizes EWS::FLI1, FLI1, and ERG. (**a**) Immunoblot analysis showing FLI1 1.2 monoclonal antibody (1:1000 dilution) detection of EWS::FLI1 fusion protein (15 μg of total protein) in various ES cell lines with EWS::FLI1 fusion type 1 (TC32, TC71, A673 and CHLA-10), type 2 (STA.ET.7.2, SKES), and type 3 (A4573). CAPAN-1 lacks EWS::FLI1. (**b**) Immunoblot analysis showing ETS proteins transfected into COS7 cells and detected using anti-FLAG antibody (top) or FLI1 1.2 monoclonal antibody (middle). EWS::FLI1 was transfected without a FLAG tag. (**c**) Competition of immunoblot detection using FLI1 1.2 in the ES cell line TC32 or A4573. HEK293 lack EWS::FLI1. FLI1 1.2 was mixed with 100 nM peptide-1 (middle) or peptide-4 (right). (**d**) Competition of immunohistochemical analysis of cell lines A4573 and TC32 using antibody FLI1 1.2. Titrated competitive inhibitor peptide-1 was added with antibody. Images are taken at 60× on Nikon SoRa Spinning Disk Microscope with the scale bar of 10 μm. (**e**) Quantification obtained after imageJ2 analysis of A4573 representative images of 5 cells for each condition. Each dot represents the value of one cell tested (* *p* < 0.05). (**f**) Reduction of EWS::FLI1 with IPTG induced shRNA. Cells were stained with FLI1 1.2 showing the EWS::FLI1 (green) immunofluorescence expression in the presence or reduction of EWS::FLI1 in the IPTG-inducible TC32 cells. Corresponding immunoblots showing the EWS::FLI1 expression levels on the right. For all immunoblots, beta-actin was used as a loading control.

FLI1 1.2 detected all three of the most common EWS::FLI1 fusion types (type 1, exon 7/6: TC32, TC71, A673 and CHLA-10 at 68 kDa; type 2, exon 7/5: STA-ET-7.2 and SKES at 70 kDa; and type 3, exon 10/6: A4573 at 75 kDa) (Figure 3a). Pancreatic adenocarcinoma cell line CAPAN-1, lacking EWS::FLI1, did not show a signal. The antibody also detected wild-type FLI1 in Acute Lymphoblastic Leukemia (ALL) MOLT-4 cells (Figure S3a). We singly transfected FLAG-tagged FLI1, ERG, ETV1, and ETV5 into COS7 cells and showed FLI1 1.2 detection of only FLI1 and ERG. ERG and FLI1 demonstrate 75% homology in the region of the immunizing peptide sequence. However, ETV1 and ETV5, other members of the ETS family of proteins (Figure S3b), have no significant homology with the immunizing peptide (epitope) and thus, are not detected by FLI1 1.2 (Figure 3b).

Cellular validation of FLI1 1.2 was performed in two ES cell lines, A4573 (type 3) and TC32 (type 1). FLI1 1.2 detected EWS::FLI1 in immunoblots for these two cell lines. The detection was competed away by peptide-1, while peptide-4 did not compete away the signal (Figure 3c). Fixed coverslips from each cell line were stained with immunopurified FLI1 1.2, along with a titration of competing peptide-1. Both cell lines showed strong nuclear staining for EWS::FLI1, which was confirmed by DAPI overlay (Figure 3d). Titration of peptide-1 competed away the EWS::FLI1 nuclear signal between 12.5 and 125 nM peptide addition, which corresponded to a ratio of 1:10-1:1 with FLI1 1.2. (Figure 3d,e). Fluorescence microscopy demonstrated IPTG-inducible EWS::FLI1 knockdown showed diminished EWS::FLI1 (green) signal compared to WT cells, and their corresponding immunoblot showed reduction of EWS::FLI1 using FLI1 1.2 in TC32 cells (Figure 3f).

### 3.5. Proteins in Complex with EWS::FLI1 Are Identified by Both Biochemical and Cellular Methods

**Figure 4:**
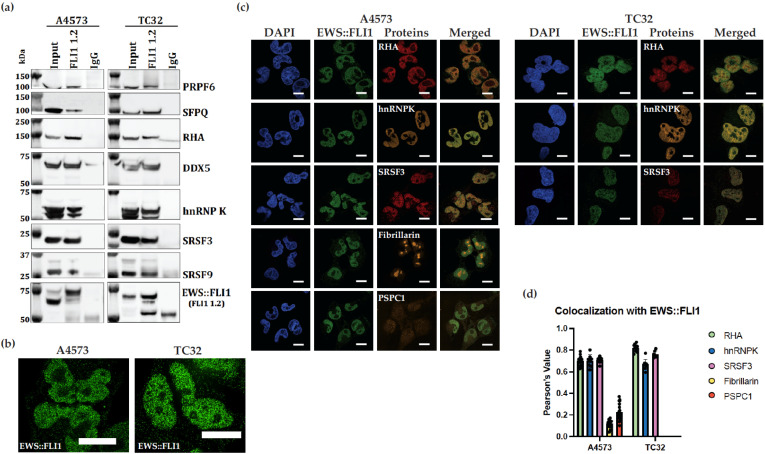
Immunopurified FLI1 1.2 antibody demonstrates effective EWS::FLI1 and protein partner co-immunoprecipitation and colocalization. (**a**) Nuclear protein lysate from ES A4573 and TC32 was mixed with FLI1 1.2 (or mouse IgG) to co-immunoprecipitate EWS::FLI1 and various protein partners. Immunoblots for each protein are indicated on the right. An amount of 10% of total nuclear lysate was used as input. (**b**) ES A4573 and TC32 IF with FLI1 demonstrating a punctate pattern of discrete foci. (**c**) Immunofluorescence of ES cell lines stained with FLI1 1.2 and indicated protein antibodies for either RHA, hnRNPK, SRSF3, fibrillarin, or PSPC1. Cells were imaged at 60× on a Nikon SoRa Spinning Disk Microscope (Scale bar is 10 μm). Merged channels (yellow) show colocalization with EWS::FLI1. (**d**) Quantified colocalization of proteins was calculated using Pearson’s test. The proteins RHA, hnRNPK, and SRSF3 showed colocalization, *p* < 0.05. Each dot represents the value of one cell tested.

We used two ES cell lines, A4573 and TC32, with two of the translocation types described above. We readily identified complexed proteins in solution with EWS::FLI1, many of which were previously reported by us or others (Figure 4a). Unlike prior studies, we did not pretreat lysates with RNase, as our goal was simply to see if FLI1 1.2 would co-IP key EWS::FLI1-interacting proteins. One of our goals in creating a monoclonal antibody was to address our inability to obtain successful co-IP with commercially available antibodies. Several commercial FLI1 antibodies were tested (Abcam, Waltham, MA, USA, Cat. No. ab15289 and MyBioSource, San Diego, CA, USA; Cat. No. MBS301248). The Abcam (Waltham, MA, USA,) antibody, which is derived from rabbit serum and is polyclonal, was ineffective in binding to EWS::FLI1 in solution in order to precipitate it; no co-IP protein of RHA was detected (Figure S4a,b). The MyBioSource antibody performed satisfactorily in binding to EWS::FLI1 in solution; however, it did not co-IP RHA (Figure S4c,d). For comparison, we demonstrated that FLI1 1.2 was able to co-IP EWS::FLI1 and RHA, as well as many of its other known protein partners, including PRPF6, SFPQ, DDX5, hnRNPK, SRSF3, and SRSF9, in two cell lines (Figure 4a).

Identifying complexes in cells has both the advantage of validating the interaction as well as showing which compartments of the cell contain the complex. High-resolution imaging captured EWS::FLI1 in a punctate pattern consistent with hubs or biomolecular condensates (Figure 4b), but these experiments were not designed to address the biomolecular condensates question [32,33,34,35]. Neither FLI1 nor ERG is expressed in ES cells, so imaging is most likely specific for EWS::FLI1 staining [36,37]. Using a Nikon SoRa spinning disk microscope and FLI1 1.2, we evaluated EWS::FLI1 in complex with various proteins, including RHA, hnRNPK, SRSF3, fibrillarin, and PSPC1. FLI1 1.2 effectively identified EWS::FLI1 in the nucleus, along with its known protein partners RHA, hnRNPK, and SRSF3 in two cell lines (Figure 4c). Fibrillarin and PSPC1 are used as markers for nucleoli and paraspeckles, respectively, both of which are not known to colocalize with EWS::FLI1 (Figure 4c). Our imaging and subsequent colocalization analysis was consistent with this expectation, as neither of these proteins demonstrated statistically significant Pearson’s values of colocalization with EWS::FLI1 (Figure 4c,d).

### 3.6. scFv Generated from Monoclonal Antibody FLI1 1.2 Recognizes and Can Degrade EWS::FLI1 Expressed in HEK 293Ts

**Figure 5:**
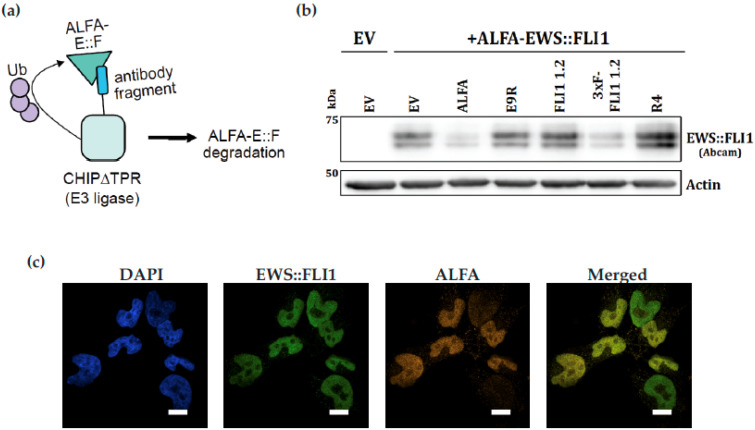
scFv generated from FLI1 1.2 can recognize overexpressed EWS::FLI1 in HEK 293Ts and degrade it when fused to an E3 ligase. (**a**) Schematic of the dTAG approach used to study a codon-optimized single-chain variable fragment (scFv) version of FLI1 1.2 fused to CHIP∆TPR, a ubiquitin E3 ligase fragment. (**b**) Various targeting modules were fused to the E3 ligase fragment CHIP∆TPR and co-transfected with ALFA-tagged EWS::FLI1 into HEK293T cells. Targeting modules are anti-ALFA tag nanobody (ALFA) and a 3xFLAG-tagged single-chain variable fragment generated from the FLI1 1.2 (3xF-FLI1 1.2), FLI1 1.2 scFv without an N-terminal 3xFLAG tag (FLI1 1.2), peptide E9R, and beta-galactosidase (R4). (**c**) ALFA-EWS::FLI1 expressed in 293T cells with simultaneous IHC using FLI1 1.2 (green) and ALFA antibody (orange). Merged images in yellow. Images are taken at 60× on Nikon SoRa Spinning Disk Microscope (Scale bar is 10 μm).

In order to create a system that allows for the selective degradation of EWS::FLI1, we generated a codon-optimized single-chain variable fragment (scFv) version of FLI1 1.2. We fused the scFv to CHIP∆TPR, an ubiquitin E3 ligase fragment, to generate a protein-based degrader of EWS::FLI1 (Figure 5a). We co-transfected HEK293T cells with ALFA-tagged EWS::FLI1 and either an empty vector, an anti-ALFA nanobody-based degrader (ALFA), our scFv-based degrader (FLI1 1.2), a 3xFLAG-tagged version of our degrader (3xF-FLI1 1.2), or a beta-galactosidase scFv-based degrader (R4). We show that degrader 3xF-FLI1 1.2 is able to reduce levels of exogenous EWS::FLI1 with a similar effect as a degrader targeting the ALFA epitope tag (Figure 5b). The negative control targeting beta-galactosidase did not affect levels of EWS::FLI1 in this system. Using a Nikon SoRa spinning disk microscope, FLI1 1.2 was able to effectively identify the nuclear localization of both endogenous (green) and ALFA-tagged exogenous EWS::FLI1 (orange) in A4573 ES cells. The exogenous ALFA-tagged EWS::FLI1 (orange) was detected with the ALFA antibody, and an overlay shows a similar pattern of staining (Figure 5c).

### 3.7. FLI1 1.2 Not Useful in Paraffin-Embedded Tissues

In order to comprehensively evaluate the abilities of FLI1 1.2 monoclonal antibody to detect EWS::FLI1, we stained normal human tonsil and a TC32 ES xenograft from a prior study. We evaluated a commercial FLI1 antibody that showed a nuclear signal in human tonsil, but with an unremarkable signal-to-noise ratio (Figure S5a). FLI1 1.2 showed weak nuclear staining in human tonsil (Figure S5b). However, when the ES xenograft was stained, it showed very strong CD99 staining [38]. In contrast, with FLI1 1.2, very little nuclear signal was observed (Figure S5c).

## 4. Discussion

This study introduces a novel FLI1 monoclonal antibody that detects the EWS::FLI1 fusion oncoprotein in ES. We show that the antibody has relative specificity for ETS family members FLI1 and ERG. Competitive peptide experiments in multiple platforms demonstrate epitope-specific recognition. FLI1 1.2 is able to recognize EWS::FLI1 in complexes with other proteins in both biochemical IP as well as intranuclear IF assays. Finally, this antibody provides an opportunity to degrade EWS::FLI1 in order to better study its function.

The epitope was selected based on an antigenicity algorithm that predicts a successful antibody. The chosen antibody recognition site is the one closest to the DNA-binding domain. This region has been previously identified as having some tertiary structure [39], unlike the distal regions, which are more intrinsically disordered [35,40]. FLI1 2.1 and 2.5 hybridomas have high ELISA titers, but neither clone detected the EWS::FLI1 fusion oncoproteins by immunoblot in both A4573 and TC32 cell lines (Figure S1b). The epitope-4 region of the antibody performed reasonably well with the co-IP, but was not selected based on the initial lower ELISA titer. FLI1 4.6 could be further evaluated in the future.

Co-immunoprecipitation is a critical method for studying the EWS::FLI1 proteome to understand the mechanistic functions of oncogenesis with its protein partners. Many commercial FLI1 antibodies are not successful in the co-IP of proteins to recognize complexes. Immunoprecipitation with our FLI1 1.2 monoclonal antibody successfully captured EWS::FLI1 fusion proteins as well as critical protein binding partners, including some spliceosome complex proteins [9]. We demonstrated that FLI1 1.2 is capable of detecting EWS::FLI1 complexes with many splicing and RNA metabolism proteins (Figure 4a). Since this antibody is derived from a renewable hybridoma clone, it should provide a continuous source for studying EWS::FLI1 interactions.

The ability to localize EWS::FLI1 in cells is important for understanding its function. We tested FLI1 1.2 using confocal microscopy, showing clear EWS::FLI1 nuclear localization and a punctate pattern of discrete foci in ES [33]. Our data support interactions in these punctate structures, including SRSF3 and hnRNPK (spliceosome) and RHA (transcriptional complexes), while specifically not interacting with paraspeckle or nucleolar proteins. Many prostate cancers carry a translocation leading to the TMPRSS2::ERG oncoprotein leading to amplified ERG levels which could potentially be detected by FLI1 1.2 [41].

This antibody and the hybridoma clone also provide clues to the specific interactions of EWS::FLI1 through our ability to control its degradation. These contributions will allow for the development of future reagents that can further elucidate EWS::FLI1 protein complex formation and biomolecular condensate synthesis.

## 5. Conclusions

These experimental data confirm that FLI1 1.2 provides utility for studying EWS::FLI1, and we highlight multiple applications of this antibody to support its broader utility in enabling further research. This reagent will provide the research community with a valuable tool for further biochemical and genomic interrogation of oncogenesis. Further knowledge of this enigmatic protein holds promise for future therapeutic opportunities for patients who develop ES.

## Data Availability

The data presented in this study are available on request from the corresponding author (S.P.S. and J.A.T.).

## Supplementary Materials

The following supporting information can be downloaded at: https://www.mdpi.com/article/10.3390/antib14040097/s1, Figure S1: FLI1 epitopes for the custom monoclonal antibody production and their validations; Figure S2: AlphaFold3 to predicts structure of heavy and light chains in complex with FLI1 1.2 epitope; Figure S3: FLI1 1.2 detects wild-type FLI1 in ALL MOLT-4 cells; Figure S4: Current commercial FLI1 antibodies do not support EWS::FLI1 protein partner precipitation; Figure S5: FLI1 1.2 did not demonstrate a strong signal to noise ratio in paraffin embedded ES tissue.
