# Supplementary material for: A Novel FLI1 Monoclonal Antibody Which Recognizes EWS::FLI1 with High Affinity Is Useful for Detecting Ewing Sarcoma
Source: Antibodies (Basel). 2025 Nov 10;14(4):97. doi: 10.3390/antib14040097 (PMC12641858; doi:10.3390/antib14040097)
Supplement: Supplementary file 1 [file antibodies-14-00097-s001.zip › antibodies-3870038-supplementary.pdf]

(a)

Target protein	The Epitope Identification/ Peptide sequence	Elisa Detection limit (ng)	Custom Label	Final selection
FLI1	SSMYKYPSDISY	0.05	1.1	✓
		0.05	1.2	✓ *
		0.25	1.3	
		0.01	1.4	✓
		0.25	1.5	
		1	1.6	
	ISYMPSYHAHQQ	0.01	2.1	
		0.05	2.2	
		5	2.3	
		1	2.4	
		0.01	2.5	
		1	2.6	
	HQQKVNFVPPHP	0.25	3.1	
		1	3.2	
		0.25	3.3	
		0.05	3.4	
		0.05	3.5	
	NPNVPRHPNTHV	1	4.1	
		0.05	4.2	
		0.25	4.3	
		0.05	4.4	
		0.25	4.5	
		0.25	4.6	✓
		5	4.7	

(b)

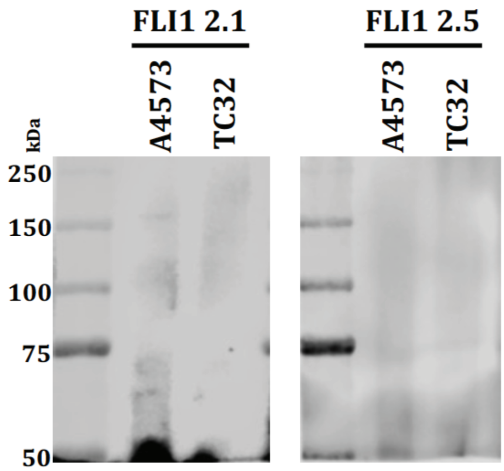

Figure S1. FLI1 epitopes for custom monoclonal antibody production and their validations. **(a)** Twenty-four hybridoma clones were generated based on the four-peptide sequence and their validations of ELISA with the detection limits are shown. Check mark indicates the selected hybridoma clones for initial western blot analysis. *Indicates the hybridoma FLI1 1.2 finally used for mass monoclonal antibody generation and purification. **(b)** Immunoblot analysis showing selected custom FLI1 monoclonal antibody (1:1000 dilution) detection of EWS::FLI1 fusion protein (15 µg of total protein) in the ES cell line TC32 (type 1) and A4573 (type 3).

(a)

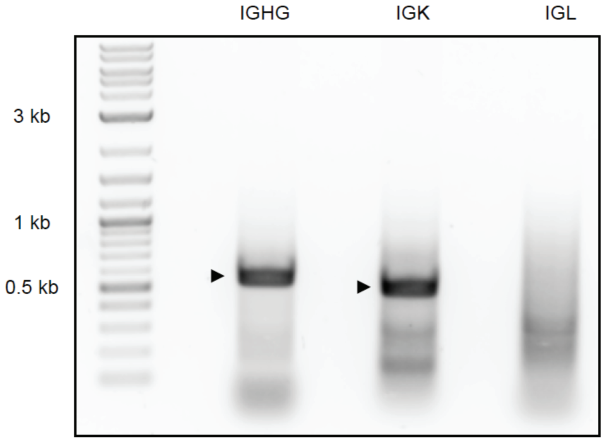

(b)

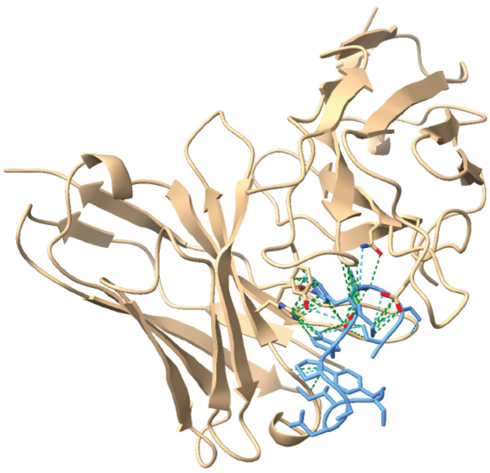

Figure S2. AlphaFold3 to predicts structure of heavy and light chains in complex with FLI1 1.2 epitope. (a) Variable regions of heavy and light chains were reverse transcribed from RNA extracted from hybridoma clone FLI1 1.2 and amplified for gel extraction and sequencing. Amplicons were detected when using primers specific for the heavy chain and the light kappa chain shown with arrow head. **(b)** AlphaFold3 predicted a structure of the heavy and light chains of antibody FLI1 1.2 (tan) with the epitope peptide (blue). ChimeraX software indicated several potential hydrogen bonds (light blue) and hydrophobic contacts (green) between the heavy and light chains and the peptide.

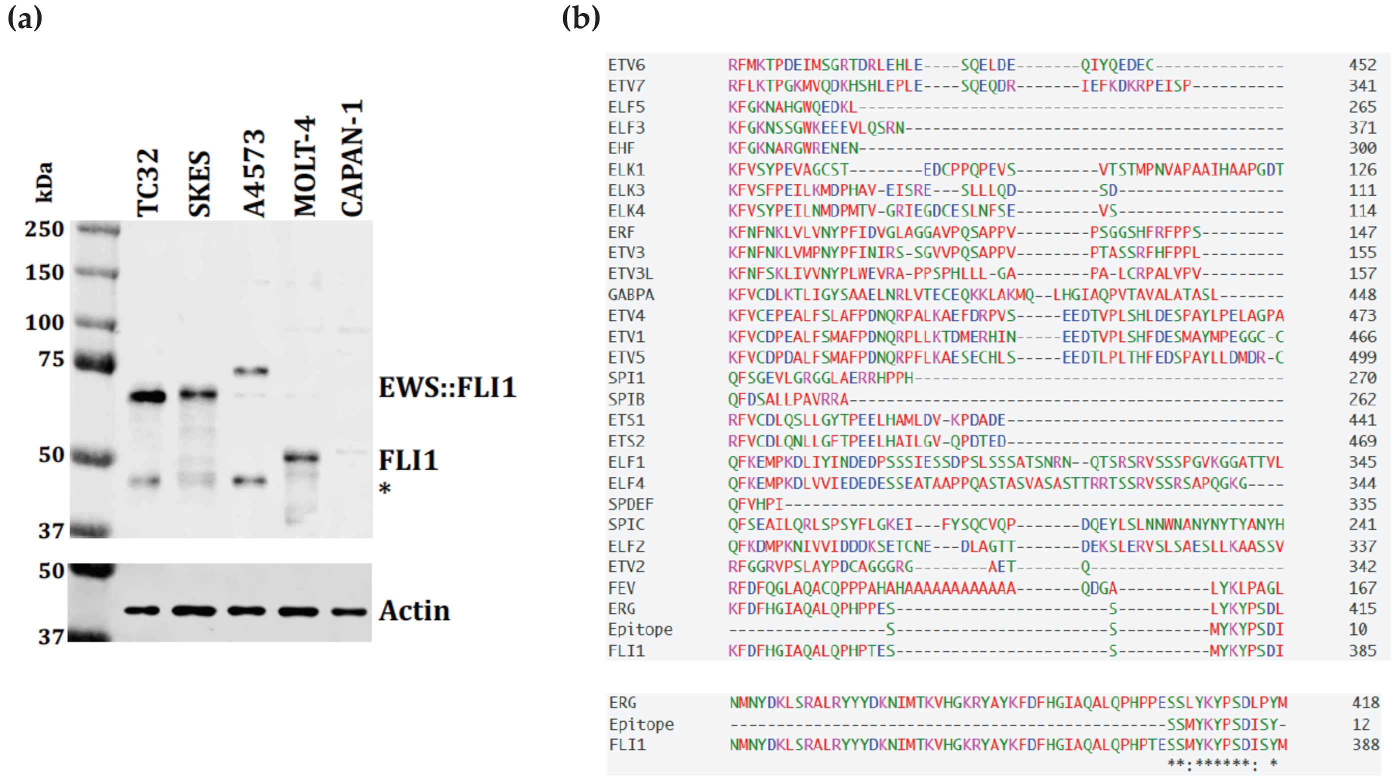

Figure S3. FLI1 1.2 detects wild-type FLI1 in ALL MOLT-4 cells. (a) Immunoblot showing wild-type FLI1 detection using FLI1 1.2 in the ALL MOLT-4 cells and EWS::FLI1 fusion protein detection in the ES cell lines with EWS::FLI1 fusion type 1 (TC32), type 2 (SKES) and type 3 (A4573). CAPAN-1 lacks EWS::FLI1. Actin was used as a loading control. *Indicates the non-specific bands. **(b)** Sequence alignment for the 28 paralogous human ETS transcription factors with custom FLI1 1.2 epitope sequence (Epitope) using Clustal Omega multiple sequence alignment (The EMBL-EBI Job Dispatcher sequence analysis tools framework in 2024). Proteins were identified by UniProtKB protein name and residue numbers. Residues are colored by amino acid types. Asterisks denote positions with amino acid identity.

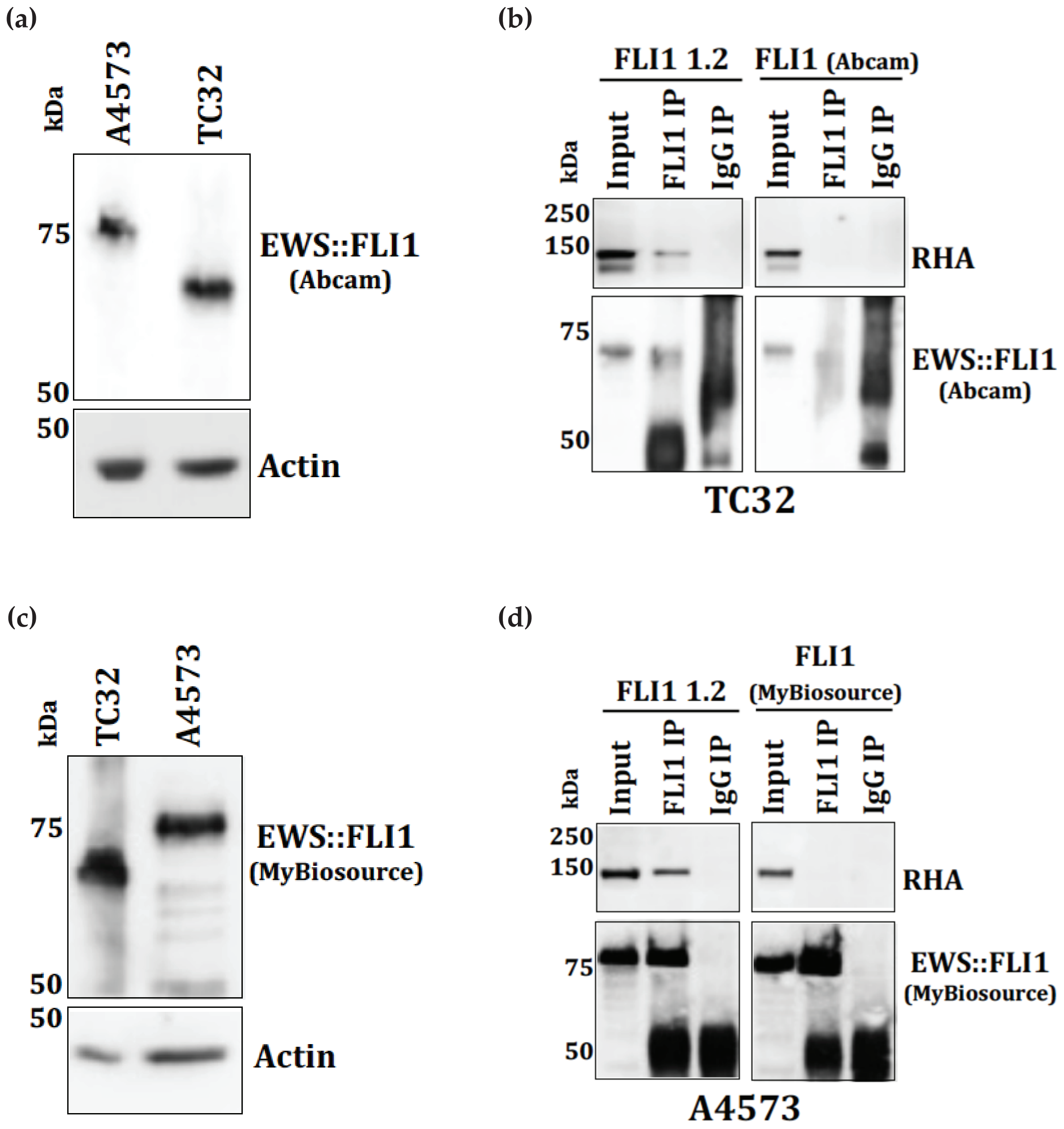

Figure S4. Current commercial FLI1 antibodies do not support EWS::FLI1 protein partner precipitation. **(a)** Immunoblot analysis showing EWS::FLI1 fusion protein detection using FLI1 commercial antibody 1 (Abcam Cat. No. ab15289) in the ES cell line TC32 (type 1) and A4573 (type 3). **(b)** Nuclear co-IP of EWS::FLI1 using custom FLI1 1.2 monoclonal antibody (Left) and FLI1 commercial antibody 1 (Abcam Cat. No. ab15289) (Right). 10% of total nuclear lysate was used as input. Immunoblotting for precipitated protein RHA. Mouse monoclonal IgG antibody was the antibody control. **(c)** Immunoblot analysis showing EWS::FLI1 fusion protein detection using FLI1 commercial antibody 2 (MyBiosource Cat. No. MBS301248) in the ES cells. **(d)** Nuclear co-IP of EWS::FLI1 using custom FLI1 1.2 monoclonal antibody (Left) and FLI1 commercial antibody 2 (MyBiosource Cat. No. MBS301248) (Right). 10% of total nuclear lysate was used as input. Immunoblotting for precipitated protein RHA. Mouse monoclonal IgG antibody was the antibody control.

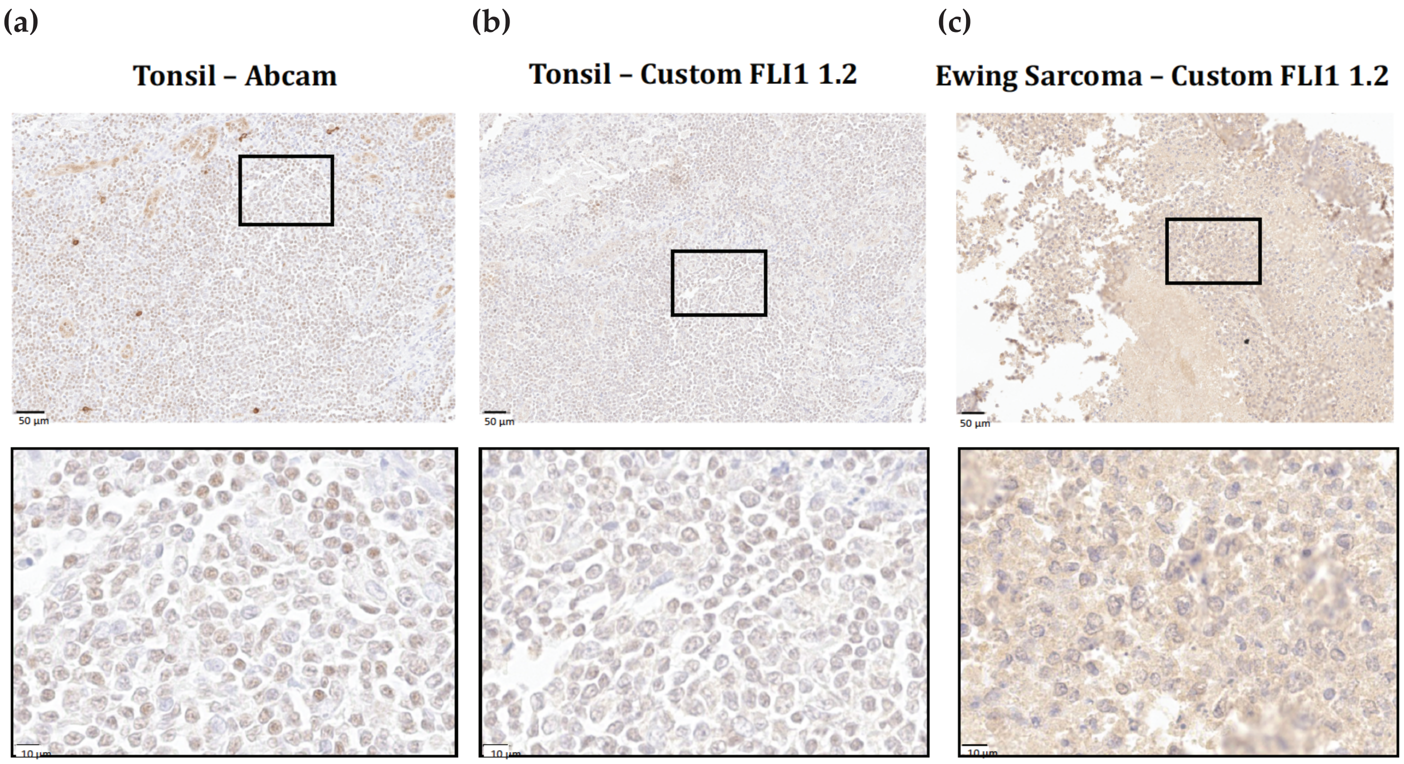

Figure S5. FLI 1.2 did not demonstrate a strong signal to noise ratio in paraffin embedded ES tissue.
(a) Human tonsil stained with FLI1 commercial antibody (1/100, Abcam Cat. No. ab15289). **(b)** Human tonsil stained with FLI1 1.2, 1/100 dilution. **(c)** ES xenograft stained with FLI1 1.2, 1/100 dilution.
